# Exploring psychological tradeoffs: Developing and demonstrating an R Shiny app for Pareto optimization

**DOI:** 10.3758/s13428-026-03093-y

**Published:** 2026-06-30

**Authors:** Yixiao Dong, Deodatta Baral, Kushmakar Baral, Denis Dumas

**Affiliations:** 1 Department of Education, Gevirtz Graduate School of Education, University of California, Santa Barbara, CA 93106-9490 USA; 2 University of Denver, Denver, CO USA; 3 University of Georgia, Athens, GA USA

**Keywords:** Optimization, R Shiny app, Tradeoffs, Pareto frontier, Multi-objective

## Abstract

While some are neutral, many psychological constructs (e.g., depression, learning motivation, or antisocial behavior) carry clear directional expectations that align with social or ethical principles and values. When a construct is framed with the goal of moving toward its socially desirable direction, it becomes a meaningful psychological objective to pursue. People pursue different objectives in their daily lives, sometimes simultaneously. During this process, tradeoffs occur when objectives are in tension or conflict (e.g., speed and accuracy in problem-solving), meaning they cannot be consistently improved without compromising one another. While certain psychological tradeoffs have been well studied, others remain underexplored or possibly even unidentified. One critical reason is that mainstream analytic methods used in psychological research are not designed to investigate such tradeoffs. Fortunately, a suitable method has long existed in other disciplines. Pareto optimization (PO) is an effective analytic framework widely applied in fields such as biology, economics, and engineering to investigate tradeoffs among multiple competing objectives. In this tutorial, we review the core conceptual and methodological foundations of PO and aim to bring this classic method to a psychological audience. Moreover, we develop a user-friendly R Shiny application (named PO-Run) for conducting PO analyses and adapt the Marginal Rate of Substitution Index from econometrics to quantify psychological tradeoffs. The application can be accessed via https://paretooptimization.shinyapps.io/Pareto/, and its utility is further illustrated through a real-world psychological example. Methodological considerations, guidance for using results, and future directions for advancing the PO method are discussed.

## Introduction

Psychological constructs (e.g., the personality trait of openness) can be mainly value-neutral, as higher or lower levels do not necessarily lead to positive outcomes in human life. In contrast, other constructs (e.g., depression, learning motivation, or antisocial behavior) carry clearer directional expectations that align with social or ethical principles and values. When a construct is framed with the goal of moving toward its socially desirable direction, it becomes a meaningful *psychological objective* to pursue. People pursue different objectives in their daily lives, sometimes simultaneously. During this process, *tradeoffs* occur when those objectives are in tension or conflict, meaning they cannot be consistently improved without compromising one another.

As an example, we kindly ask readers to observe the following array of numbers: 7, 8, 3, 4, 6. Take your time to find the median. We believe it is relatively easy to identify *6* as the median after a period of thinking. Now, please observe another number array and try speaking out the median as quickly as possible: 7, 2, 5, 8, 9. This time, anyone could have easily made a mistake when rushing to find the median 7. In solving many daily tasks, the accuracy of our decisions can be negatively impacted by the speed at which those decisions are made, because speed and accuracy manifest a well-established tradeoff relation (e.g., Bogacz et al., 2010; Dickman & Meyer, 1988; Heitz, 2014).

In psychology, some tradeoffs among constructs, such as between originality and appropriateness in creative ideation (Dumas et al., 2025), between life-goal investment and positive emotion (Pomerantz et al., 2000), and between achievement motivation in school and the social cost of studying (Jiang et al., 2018), have been observed and documented. While certain types of psychological tradeoffs (e.g., speed–accuracy) have been well studied, many remain underexplored. Possibly, more psychological tradeoffs still remain to be discovered. One critical reason is that mainstream analytic methods in psychology, ranging from basic mean comparisons to more advanced statistical modeling, are not specifically designed to investigate tradeoff relations. Many attempts to investigate psychological tradeoffs have largely relied on relational analyses that provided either direct evidence (e.g., the inverted-U relation between decision time and error rate; Bogacz et al., 2010) or indirect evidence that requires further interpretation and theorizing by psychologists (e.g., high goal investment resulted in both positive and negative emotional consequences; Pomerantz et al., 2000). Among these attempts, the methodologies for grounding the scientific evidence base for tradeoffs can vary substantially.

Fortunately, a suitable method has long existed in other disciplines: Pareto optimization (PO), which is a classic analytic framework originally developed by Vilfredo Pareto in the early 20th century (1906/1971). As one example of how this method has dovetailed with important theoretical issues in another field, consider that Charles Darwin’s monumental work *The Origin of Species* (1859/1964), described species’ trait adaptations to different environments as evidence of biological tradeoffs. Later, biologists have worked to strengthen the empirical evidence base supporting Darwin’s observations (Agrawal et al., 2010). Among these endeavors, Pareto optimization has repeatedly shown its effectiveness (e.g., Warmflash et al., 2012). Beyond biology, economics (e.g., Buchanan, 1962) and engineering researchers (e.g., Marler & Arora, 2004) have also frequently employed PO for balancing multiple competing objectives and investigating tradeoffs. But despite its popularity and utility in other fields, PO is scarcely applied in psychology. Given that PO can accommodate both latent and directly observable psychological constructs, this approach holds considerable potential for psychological research.

We believe that this underutilization may be explained by two key challenges. First, a conceptual explanation is needed as to how PO can be most effectively applied in psychological research for identifying and examining tradeoffs. Second, the implementation of PO is not readily accessible to the psychology research community, who may be less familiar with PO-associated analysis and coding. The current study thus aims to address these challenges by translating the PO approach into psychological research and improving its accessibility by offering a user-friendly R Shiny application: *PO-Run*. Specifically, we start with a brief review of the core conceptual and mathematical foundations of PO and then discuss their connections to the investigation of psychological tradeoffs. In addition, we illustrate the use of the application with a real-world empirical example in psychology, which examines the plausible tradeoffs between promoting *critical actions* (i.e., actions taken to enact sociopolitical changes, Diemer et al., 2022) and minimizing *mental stress* (which tends to unfortunately increase as individuals take more critical action). Along with the illustration, we also provide several methodological considerations to guide researchers in effectively applying this approach in future studies.

Psychological tradeoffs can be studied through analyses with inter- and intra-individual foci, as is the case with many other investigations of psychological phenomena, and findings from one level do not directly inform the other except under restrictive assumptions (Molenaar, 2015). The present work follows the standard statistical analysis framework in approaching tradeoffs as a population-level phenomenon, that is, analyzing *inter-individual* data from a sample of subjects and generalizing the findings to a targeted population. Accordingly, the results of the present method with an inter-individual focus should not be taken as strong theoretical conclusions about intra-individual tradeoff dynamics, and vice versa.

## Pareto optimization

**Fig. 1:**
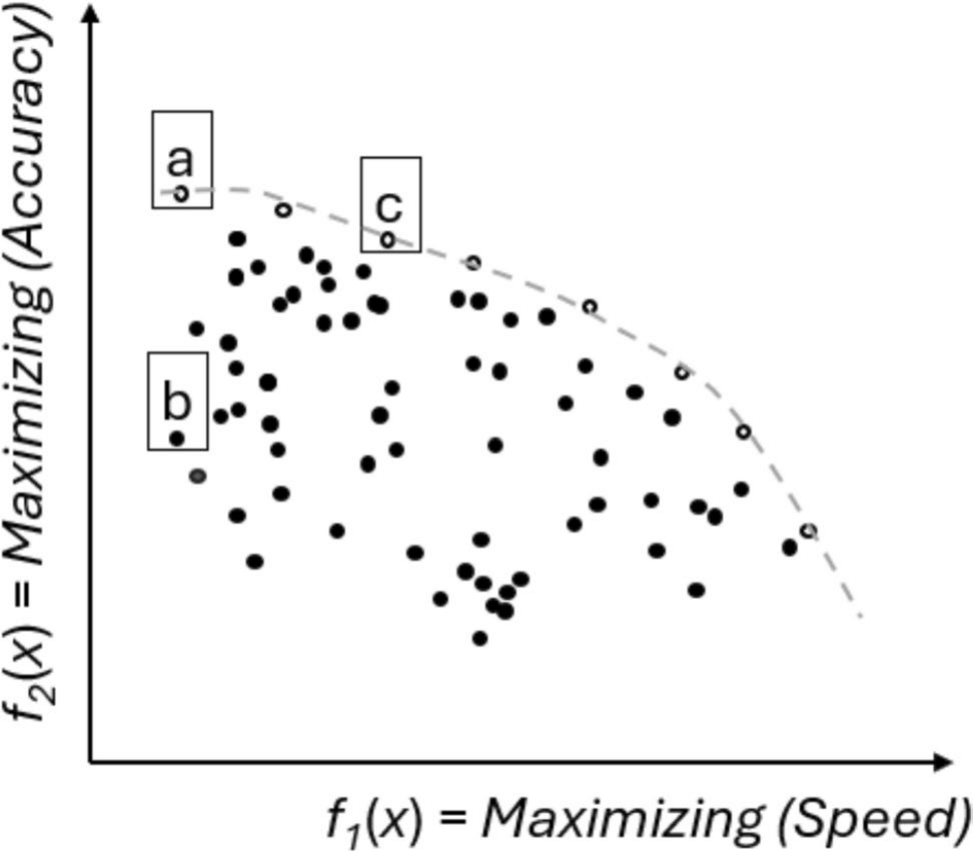
Conceptual representation of two-dimensional Pareto optimization

In cases where multiple objectives are in conflict (i.e., they cannot be maximized at the same time), Pareto optimization exhausts the potential for concurrent improvement among objectives (Marler & Arora, 2004). As a consequence, it can identify instances in a dataset that allow each objective to be achieved as much as possible, given the tradeoff. Although PO can technically be applied to a single optimization objective, it is quintessentially useful when two or more objectives are involved. Tradeoffs inherently represent conflicts among objectives, where improving one requires sacrificing another. As shown at the beginning of the article, a classic example in cognitive psychology is the speed–accuracy tradeoff in decision-making (Pachella, 1974). It describes how individuals tend to make more errors when prioritizing speed, whereas improving accuracy typically requires slowing down the decision-making process (Shaddy et al., 2021). The Pareto-optimal points or solutions represent conditions at which accuracy cannot be further improved without reducing speed, and vice versa (shown as the empty dots in the conceptual Fig. 1).

### Defining optimization objectives and functions

Each of the *k* PO objectives can be defined through a distinct objective function, denoted as *f*_*k*_(*x*). PO can then be expressed as a general formulation of a vector optimization problem, as in Equation 1 below:

1 $$Optimize\overrightarrow{F}(x)=[{f}_{1}(x),{f}_{2}(x),{f}_{3}(x),...,{f}_{k}(x)]$$

The complexity of each *f*_*k*_(*x*) may vary considerably depending on the specific aims of researchers using PO. In the context of examining tradeoffs, an objective function can be defined parsimoniously as *simultaneously* maximizing (or minimizing) target variables as much as possible, given that they cannot reach their theoretical maxima (or minima) without sacrificing one another. For instance, in the case of the speed–accuracy tradeoff, the objective functions can be expressed as follows:

2 $$Max\overrightarrow{F}(x)=[{f}_{1}(x),{f}_{2}(x)]$$

where

3 $$\left\{\begin{array}{c}{f}_{1}(x)=Max(Speed)\\ {f}_{2}(x)=Max(Accuracy)\end{array}\right.$$

### Dominance and the Pareto frontier

In the multi-objective optimization literature, Steuer (1989) defined *dominance* among data points and solutions as a way of conceptualizing (non-)optimality. Let *S* be the feasible criterion space containing all attainable objective-function outcomes or solutions. A solution $\overrightarrow{F}\left({x}^{*}\right)\in S$ is considered *non-dominated* (or non-inferior) if no other solution $\overrightarrow{F}\left(x\right)\in S$ is strictly better on at least one objective function (e.g., higher speed in decision-making), without being worse on other *f*_*k*_(*x*) (e.g., accuracy in decision making). Otherwise, $\overrightarrow{F}\left({x}^{*}\right)$ is not optimal and is *dominated* by other solutions.

This rule also provides the basis for understanding the relationships among multiple solutions. In Fig. 1, three candidate solutions (a, b, c) are highlighted. Based on Steuer’s rule, solution $\overrightarrow{F}\left({x}^{a}\right)$ dominates $\overrightarrow{F}\left({x}^{b}\right)$, because it comes with a higher value on the accuracy objective function (placed along the *y-*axis in Fig. 1) while holding the same value on the speed objective function (the *x*-axis in Fig. 1). Similarly, solution $\overrightarrow{F}\left({x}^{c}\right)$ dominates $\overrightarrow{F}\left({x}^{b}\right)$ by attaining higher values on both speed and accuracy objectives. As for $\overrightarrow{F}\left({x}^{a}\right)$ and $\overrightarrow{F}\left({x}^{c}\right)$, neither solution dominates the other: the former performs better on accuracy, whereas the latter performs better on speed. In this way, both points *a* and *c* in Figure 1 can be considered optimal as per the Pareto function.

Notably, non-dominated solutions and Pareto-optimal solutions are two terms often used interchangeably (Marler & Arora, 2004), which means all the empty dots previously labeled as Pareto optimality in Figure 1 are indeed non-dominated solutions. The collection of these solutions shapes a boundary known as the *Pareto frontier* (Messac et al., 2003), which is represented in the figure as a dashed curve. Applied researchers have shown great interest in the Pareto frontier and the non-dominated solutions it contains when investigating psychological tradeoffs with the PO framework (e.g., Dumas et al., 2025; Lievens et al., 2022). Across studies, identified Pareto frontiers may vary in composition because researchers can specify different numbers of Pareto layers. Pareto layers represent the hierarchical structure of solutions according to dominance depth (Avigad et al., 2010; Deb, 2011). Conceptually, the non-dominated solutions that form the Pareto frontier are located on the first layer. Solutions on the second layer are those dominated only by one or more first-layer solutions, while those on the third layer are dominated only by solutions on higher layers. In other words, Pareto layers rank solutions from the most to the least optimized and highlight the hierarchy among solutions. To obtain the most parsimonious and *exact* Pareto frontier, only the optimized solutions (i.e., the truly non-dominated) located on the first layer should be included. Selecting additional Pareto layers can be beneficial for identifying broader patterns, particularly when sample sizes are modest, and the estimated Pareto frontier may be unstable. When multiple layers are specified (e.g., 12 layers in Dumas et al., 2025), the resulting solution set extends beyond the exact Pareto frontier to include dominated solutions from lower layers, and thus these represent approximate Pareto frontiers rather than the exact frontier. A comparison between the exact and approximate Pareto frontiers is included in the later Shiny app illustration.

### Bi-objective Pareto optimization: Making PO-Run useful and parsimonious

The current PO-Run is designed to be parsimonious for its particular use of exploring psychological tradeoffs and optimizing two objectives. We acknowledge that psychological investigations are not always limited to two variables, and PO has the capacity to incorporate additional objectives and dimensions. For example, in addition to speed and accuracy, researchers may choose to optimize cognitive load in decision-making as a third objective. This elevates the problem to a multi-dimensional tradeoff that requires further multi-objective optimization. Correspondingly, an additional objective function (e.g., *f₃*(*x*) = *Min[Cognitive Load]*) would be included to achieve such an optimization goal. Nonetheless, just as bivariate correlation has served as a foundation for more complex modeling in psychological research, bi-objective Pareto optimization remains a crucial basis for understanding more complex psychological tradeoffs. Increasing the number of objectives usually comes with substantive costs, including slower search processes, greater computational demands, and challenges in visualization and interpretation. In some cases, dimension-reduction techniques (e.g., principal component analysis) have even been intentionally integrated into Pareto optimization to reduce the number of dimensions (Deb & Saxena, 2006). From this perspective, pursuing a large number of optimization objectives or dimensions is not necessarily a helpful step for investigating psychological tradeoffs. So, the method as well as the R Shiny app we present here focuses on two-dimensional instances, where the tradeoff is between two objectives.

## Digging into negatively correlated variables: Exploring plausible tradeoffs

Statistically, a negative inter-individual correlation between two generally *desired* psychological objectives serves as a useful heuristic for identifying a plausible tradeoff, since positively correlated or independent ones would reflect non-conflicting mutual growth rather than competition. It should be noticed that the presence of a negative correlation, regardless of its strength, does not in itself guarantee the existence of a genuine tradeoff (Agrawal et al., 2010). In other words, negative correlation is insufficient for justifying a tradeoff, which posits the examination of bivariate correlations among variables of interest as merely a starting point for investigation. Beyond the correlation, both theoretical grounding and additional empirical evidence are essential to justify the plausibility of a tradeoff.

### Evidence of tradeoffs from PO analysis and visualization

**Fig. 2:**
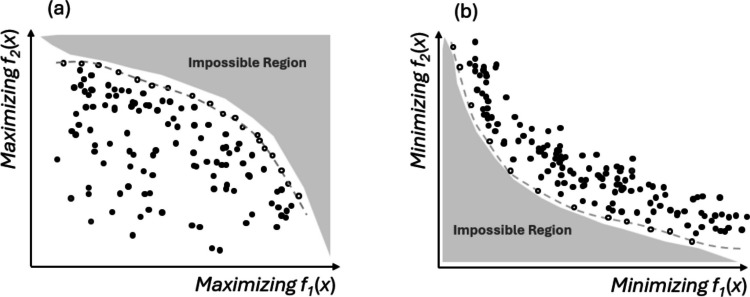
Two forms of Pareto frontier with different extents of sharpness

Visualization has been recognized as a useful and essential source of evidence for researchers to explore psychological tradeoffs with PO (e.g., Deb & Saxena, 2006; Doerner et al., 2004; Dumas et al., 2025). So, PO-Run is designed as both an analysis and a visualization tool. With two optimization objectives, the Pareto frontier typically takes the form of a curve. Whether this curve is convex or concave mainly depends on the nature of the optimization objectives. For example, the convex curve in Fig. 2a shows the case of attempting to maximize two desirable objectives, whereas the concave curve in Fig. 2b indicates an attempt to minimize two undesirable objectives. Notably, maximizing or minimizing objectives are often feasibly transferable and equalized by reversing their psychological meaning. In the decision-making example, the objectives of maximizing speed and accuracy are equivalent to minimizing time spent and error rate. However, the shape of the Pareto frontier is neither deterministic nor a definitive indicator of a tradeoff. Pareto frontiers can assume a variety of shapes (Sheftel et al., 2013; Shoval et al., 2012; Taheri et al., 2016), including a straight line^1^.

#### Pareto frontier sharpness

Instead, the *sharpness* of the frontier (Taheri et al., 2016) can serve as the key *visual* indicator of tradeoffs. However, sharpness has not been explicitly defined in the previous work. In this study, we visually evaluate the sharpness of the frontier from two facets, that is, the substantialness of the impossible region as well as the solution tightness around the frontier.

The impossible region refers to the area beyond the Pareto frontier where combinations of objective values (i.e., feasible solutions) cannot be simultaneously achieved given the inherent tradeoff constraints. In other words, no Pareto-optimized solution falls into this area. In the context of the speed–accuracy tradeoff, the impossible region would represent hypothetical combinations of very high speed and very high accuracy that no participant can achieve. Both Pareto frontiers in Fig. 2 demonstrate a clear and substantial impossible region, as no data points appear beyond the frontier within that bounded space. Figure 2a visualizes a scenario in which, at a certain point, one objective cannot be further improved without sacrificing the other (e.g., improving decision-making speed would eventually decrease accuracy), which provides evidence of a plausible psychological tradeoff. If a number of solutions fall into the upper-right corner of Fig. 2a, it would then indicate that no substantial impossible region exists, and thus, the tradeoff between speed and accuracy is not supported.

Moreover, the solution tightness around the Pareto frontier provides another visual diagnostic, which refers to the pattern of how closely feasible solutions cluster near the Pareto frontier. Tight patterns (solutions concentrated along or near the frontier) suggest that more individuals achieve near-optimal tradeoffs, while loose patterns (solutions dispersed throughout the objective space) indicate greater variability in how people navigate the tradeoff. To some extent, this is analogous to ceiling or floor effects commonly observed in psychological assessments (Keeley et al., 2013). Just as ceiling/floor effects become more pronounced when responses concentrate at scale boundaries (e.g., the first or fifth categories of a five-point Likert scale), solution tightness becomes more apparent when individuals cluster near the Pareto frontier. Within the accuracy and speed tradeoff example, solutions in Fig. 2b cluster more tightly along the Pareto frontier, producing a tighter and less scattered pattern in comparison to the pattern shown in Fig. 2a, and this may indicate a stronger tradeoff between the two conflicting objectives.

Of course, impossible regions and solution tightness can be influenced by data characteristics such as measurement error and sampling diversity. Therefore, a holistic interpretation is necessary when evaluating tradeoffs through visualization. Like other visual diagnostics (e.g., histograms or Q-Q plots for assessing normality), these patterns are most interpretable when supplemented with quantifiable metrics, even for *exploratory* purposes. Next, we adapt the marginal rate of substitution (MRS) from econometrics to quantify and explore psychological tradeoffs.

#### **Depicting psychological tradeoffs with MRS Index**

The marginal rate of substitution is a classic economic metric first introduced by Hicks and Allen (1934) and has been applied to quantify the trade-off between two competitive economic goods, such as leisure and consumption. It continues to play an important role in microeconomic theory and practice (Mas-Colell et al., 1995). Hicks and Allen’s conceptualization of MRS builds upon Pareto’s earlier work on formulating relationships between pairs of goods (Hicks & Allen, 1934), which makes the MRS and Pareto methodologies carry a natural synergy. Inherently, MRS quantifies the slope of an indifference curve, indicating the rate at which a consumer is willing to trade good A for good B while maintaining the same level of utility (Knetsch, 1989). When adapted to quantify the rate of tradeoffs between psychological objectives using Pareto optimization methods, the Pareto frontier mirrors the indifference curve while the two optimization objectives (i.e., *f*_*1*_[*x*] and *f*_*2*_[*x*]) mirror a pair of goods. The MRS_*frontier*_ can then be calculated using a finite difference approximation, based on adjacent Pareto-optimal points as the ratio of change in one objective to change in the other. Specifically, a Pareto frontier consists of *k* optimal solutions given the objectives of *f*_*1*_(*x*) and *f*_*2*_(*x*):

4 $$\{({f}_{1}^{1},{f}_{2}^{1}),({f}_{1}^{2},{f}_{2}^{2}),\dots ,({f}_{1}^{k},{f}_{2}^{k})\}$$

For each adjacent pair of points *i* and *i*+1, a discrete MRS_*i*_ representing the local tradeoff rate between the two objectives over that segment can be computed as:

5 $$MR{S}_{i}=-\frac{\Delta {f}_{2}^{i}}{\Delta {f}_{1}^{i}}=-\frac{{f}_{2}^{i+1}-{f}_{2}^{i}}{{f}_{1}^{i+1}-{f}_{1}^{i}}$$

**Fig. 3:**
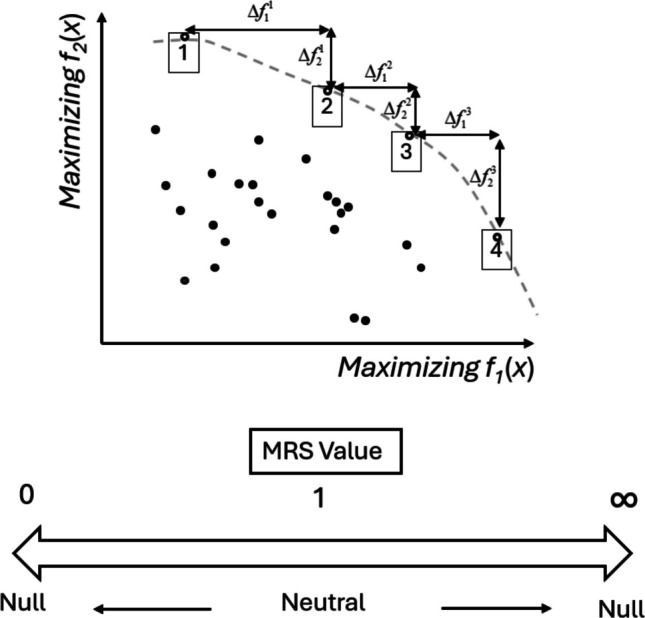
A visualization of calculating and interpreting MRS

Figure 3 displays a visualization of calculating MRS (e.g., with *k* = 4 points and segment *I* = 3). The average MRS_*frontier*_, representing the overall tradeoff rate between the two objectives, can be computed based on all *k-1* discrete MRS_*i*_ values as:

6 $$\overline{MR{S }_{frontier}}=\frac{1}{k-1}\sum_{i=1}^{i}MR{S}_{i}$$

Notably, since objectives can be on different scales, PO-run employs *z*-standardization to remove scaling effects when calculating MRS (e.g., Δz_Accuracy/Δz_Speed). In this way, the MRS estimate becomes a standardized metric that supports comparisons and interpretation. Specifically, an MRS of *m* indicates that *m* SDs of the objective on the *Y*-axis (e.g., accuracy) are sacrificed per SD of the objective on the *X*-axis (e.g., speed) gained along the Pareto frontier. Because the optimization objectives for the *X*- and *Y*-axes can be reversed, an MRS of *m* indicates the same tradeoff magnitude as an MRS of 1/*m* (e.g.,.25 and 4 both represent equal tradeoff rates). We visualized a guideline for interpreting tradeoff rate in terms of MRS, shown as a symmetric spectrum in Fig. 3. An MRS of 1 indicates a symmetric tradeoff where both objectives are equally weighted, and it is perceived as the neutral exchange. MRS values deviating from 1 in either direction indicate a higher tradeoff rate. As MRS approaches 0 or infinity, one optimization objective becomes increasingly invariant to changes in the other *across participants*, which approaches a degenerate exchange rate that constitutes a null tradeoff.

As another note, the MRS should be treated as a continuous relative index rather than a categorical classifier, so no cutoffs are specified. Moreover, a bootstrapping function has been built into PO-run to assess and visualize uncertainty in both the Pareto frontier and the MRS estimates.

### Outlining a theoretical basis for the tradeoff mechanisms: The need to align objectives

Beyond the empirical evidence (e.g., visualization and MRS estimates) garnered from Pareto optimization, a theoretical basis is essential for explaining and supporting the existence of a genuine tradeoff. Establishing a theoretical basis for a specific tradeoff requires identifying the theories that support a particular explanation of its underlying mechanism. For example, resource competition represents a typical explanation, where shared and limited resources between two constructs make it impossible to concurrently and continuously improve one objective without sacrificing another. Other explanations include motivational conflict (Miller, 1944), incommensurable values (e.g., Tetlock et al., 2000), and so forth. However, identifying appropriate theories may be just the universal component required for explaining any psychological phenomenon. For theorizing psychological tradeoffs with evidence from PO, researchers must also justify the alignment among the chosen psychological objectives for optimization. Here, *alignment* refers to both objectives holding similar value vis-à-vis human goals and ethical standards (e.g., Rao et al., 2023), meaning that people would seek to either simultaneously maximize (in the case of desirable attributes) or minimize (in the case of undesirable attributes) both objectives.

As a counter-example, if two attributes were unaligned—meaning that one was desirable (and therefore should be maximized) and the other was undesirable (and therefore should be minimized)—they would not be appropriate for the kind of analysis we are presenting in the current paper and would not indicate a genuine tradeoff in the way it is defined here. Take, for instance, the case of test anxiety (a quintessentially undesirable and distressing attribute) and test performance (a clearly desirable attribute). Test anxiety has been shown to be negatively related to test performance (Cassady & Johnson, 2002), such that students tend to perform worse when anxiety levels are high. Covington (1985) classically described the “cycle of failure,” highlighting the reciprocal negative relationship between anxiety and test performance. Other cognitive psychology studies have also shown that anxiety consumes cognitive resources and increases cognitive load (e.g., Berggren et al., 2012). Together, these findings may imitate a tradeoff mechanism: both test anxiety and cognitive task performance draw upon limited cognitive resources. Given that participants’ cognitive resources are finite, higher anxiety indirectly consumes performance, thereby producing a plausible tradeoff between these constructs.

However, such a tradeoff remains *false* when considering the alignment issue because pursuing higher test anxiety is never a meaningful or desirable objective in reality. It would be unreasonable to aim to maximize both test anxiety and performance simultaneously. Instead, alternative psychological objectives that are better aligned can be maximizing test self-assurance (or confidence) alongside performance. However, these constructs are typically positively correlated (Stankov & Lee, 2008), meaning that no genuine tradeoff exists as a starting point for psychological optimization. In sum, negative correlations among psychological objectives, empirical evidence from Pareto optimization, and theoretical mechanisms *triangulated* the plausibility of a tradeoff. In the following section, we demonstrate a tradeoff investigation via a user-friendly R Shiny application (PO-Run).

## Illustrative example

The speed and accuracy tradeoffs have been well documented in the psychological literature (e.g., Boag et al., 2023; Dickman & Meyer, 1988; Heitz, 2014), so we utilize this classic example throughout the paper to theoretically illustrate tradeoff concepts and the development of the R Shiny app. However, PO-Run is also intended for exploring not-yet-documented or understudied psychological tradeoffs, and many real-world applications may not be as clear-cut as the speed–accuracy tradeoff. Thus, we included a more novel real-world example in this section to demonstrate the use of PO-Run and the interpretation of generated results.

This real-world example used to illustrate PO-Run investigates a plausible tradeoff between critical action and mental stress. Critical action is one component of critical consciousness (CC), specifically referring to sociopolitical actions taken to address social injustice (Diemer et al., 2022). Research has shown that CC, especially the critical action domain, has been associated with different mental health issues such as depression, anxiety, and stress (Desmarais & Christophe, 2024). Individuals with higher levels of CC tend to report higher levels of stress, suggesting that the objectives of increasing CC and reducing stress may be in conflict, and a plausible tradeoff may exist.

## Transparency and openness

The present study was not preregistered. The PO-Run app can be accessed via https://paretooptimization.shinyapps.io/Pareto/. Both the annotated R codes for building the application and the data for the worked example presented here are stored on Open Science Framework (OSF): https://osf.io/6fbhu/. The open resource in the OSF repository was constructed in accordance with the recommendations of Ellis et al. (2024) and Towse et al. (2021).

## Data and measures

The data are derived from a previous study investigating vicarious exposure to racism among Asian American and African American communities (Dong, 2025). The sample consisted of 301 African American participants, 224 Asian American or Pacific Islander participants, and four multiracial (i.e., self-identified as both Asian and African American) participants. Critical action was measured using the Short Critical Consciousness Scale (Diemer et al., 2022). This subscale includes five items (e.g., “I participated in a civil rights group or organization”) rated on a five-point Likert scale (1 = *Never* to 5 = *At least once a week*). Stress was measured with the widely used Perceived Stress Scale–10 (Cohen & Williamson, 1988), which consists of 10 self-report items (e.g., “In the last month, how often have you felt nervous and stressed?”) rated on a five-point Likert scale (1 = *Never* to 5 = *Very often*). Both measures demonstrated excellent internal consistency (critical action: *α* =.90; stress: *α* =.89). To maintain the conciseness of this illustration, we calculated composite scores by simply averaging the items to represent the two constructs (Edelsbrunner, 2022). However, we acknowledge that modeling-based scoring procedures (e.g., factor analysis) may further reduce measurement error when quantifying these latent variables in real practice (McNeish & Wolf, 2020).

## Aligning the variables for optimization

The PO-Run Shiny app is set by default to maximize the variables placed on both the *X*- and *Y*-axes. However, in many applied cases, an optimization objective may require minimizing a variable value. For example, in this illustration, a higher stress level is not a desirable objective. In situations like this, researchers can simply reverse-code the variable (and even consider relabeling the objective as appropriate^2^). In our example, we reverse-coded the stress items in score calculation and labeled the construct as *peace of mind*, which allows the PO function to simultaneously maximize two desirable objectives: critical action and peace of mind. Using the terminology we introduced above, these objections are aligned because they are both desirable, and the PO function is intended to maximize them.

## PO-Run development and programming

The PO-Run application was developed in R (R Core Team, 2025) with the following packages for supporting different purposes and functions, including shiny (Chang et al., 2025) for building the interface, dplyr (Wickham, François et al., 2023) for cleaning and reshaping data, readxl (Wickham & Bryan, 2023) and haven (Wickham, Miller et al., 2023) for reading Excel, SPSS, and Stata files, ggplot2 for generating plots (Wickham, 2023), rPref (Roocks, 2016) for identifying Pareto-optimal solutions, and DT for creating interactive tables (Xie et al., 2024). A detailed and annotated version of the R codes for programming the PO-Run is stored in the OSF repository of this project.

## App input

**Fig. 4:**
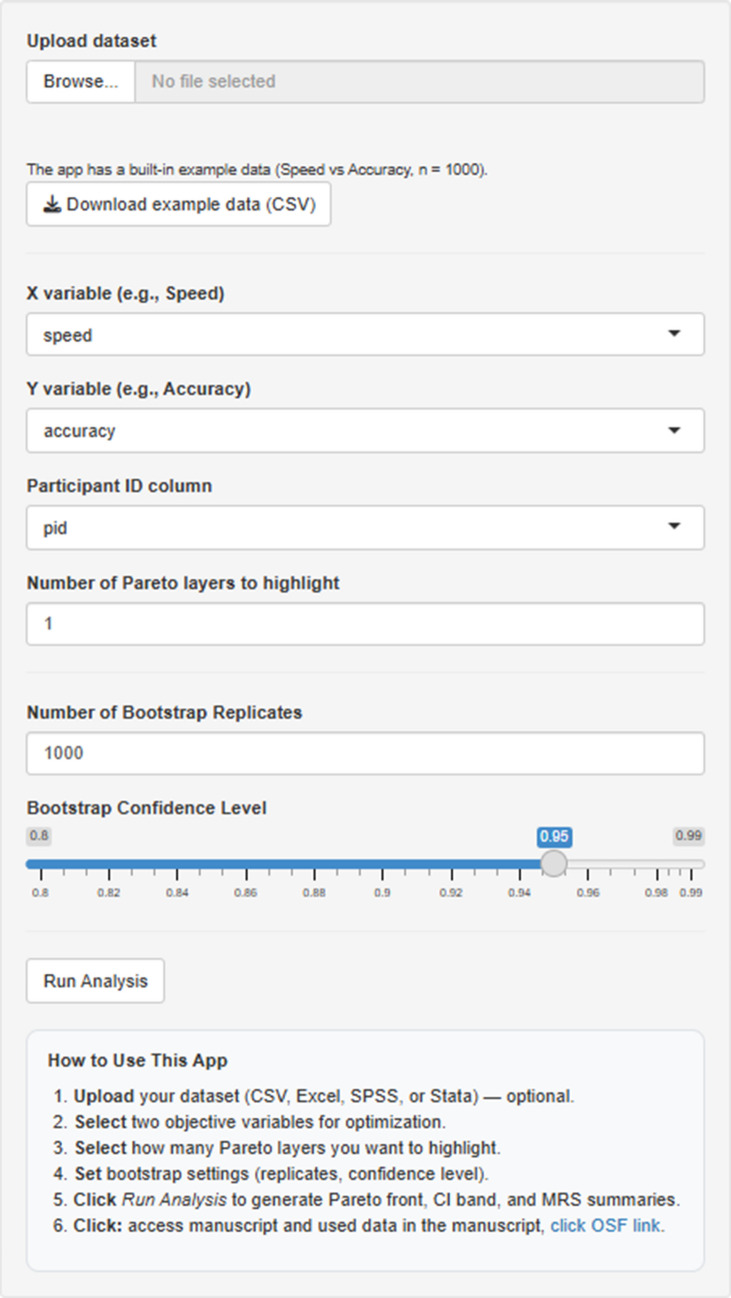

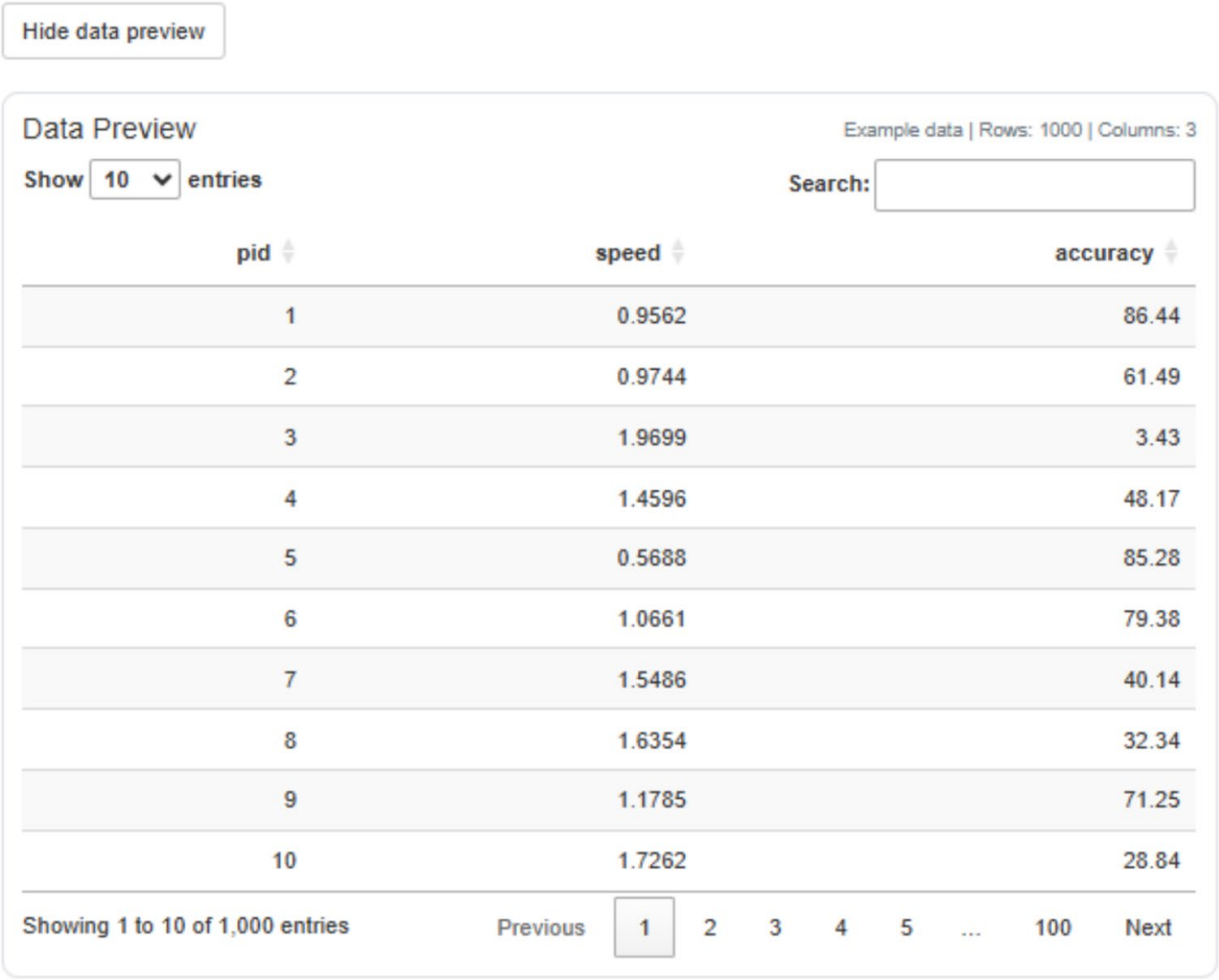
(**a**) PO-Run app input interface (**b**) PO-Run app data preview function

As shown in Fig. 4a, the application features a concise interface with stepwise instructions. The primary input involves uploading a dataset that contains the numerical variables of interest for bi-objective Pareto optimization. The app currently accepts four common data formats used in social science research: CSV (.csv), Excel (.xlsx), SPSS (.sav), and Stata (.dta). The app includes an embedded example^3^ CSV data file (Speed vs. Accuracy) to help users understand the required data format for PO-Run analysis. Users may either download this example dataset or utilize the built-in data preview function shown in Figure 4b to check data requirements.

The PO-Run app generates bootstrapped confidence intervals (CIs) for both the Pareto frontier and the MRS index. Users can customize the number of bootstrap iterations and the confidence level in terms of their research context (e.g., sample size). The conventional 95% CI and 1000 bootstrapping iterations are set as defaults, with the latter representing the standard minimum for social science applications. A larger number of iterations could result in substantially longer computation times. To provide visual feedback during analysis, we implemented a “running person” animation that indicates active computation.

Another essential configuration in PO-Run is setting the number of Pareto layers (Avigad et al., 2010), which determines the hierarchical structure of solutions according to dominance depth. By default, the app selects only the first layer, corresponding to the exact Pareto frontier of fully non-dominated solutions. As noted earlier, specifying multiple layers expands the solution set to include dominated solutions, resulting in approximate rather than exact Pareto frontiers.

## App output

**Fig. 5:**
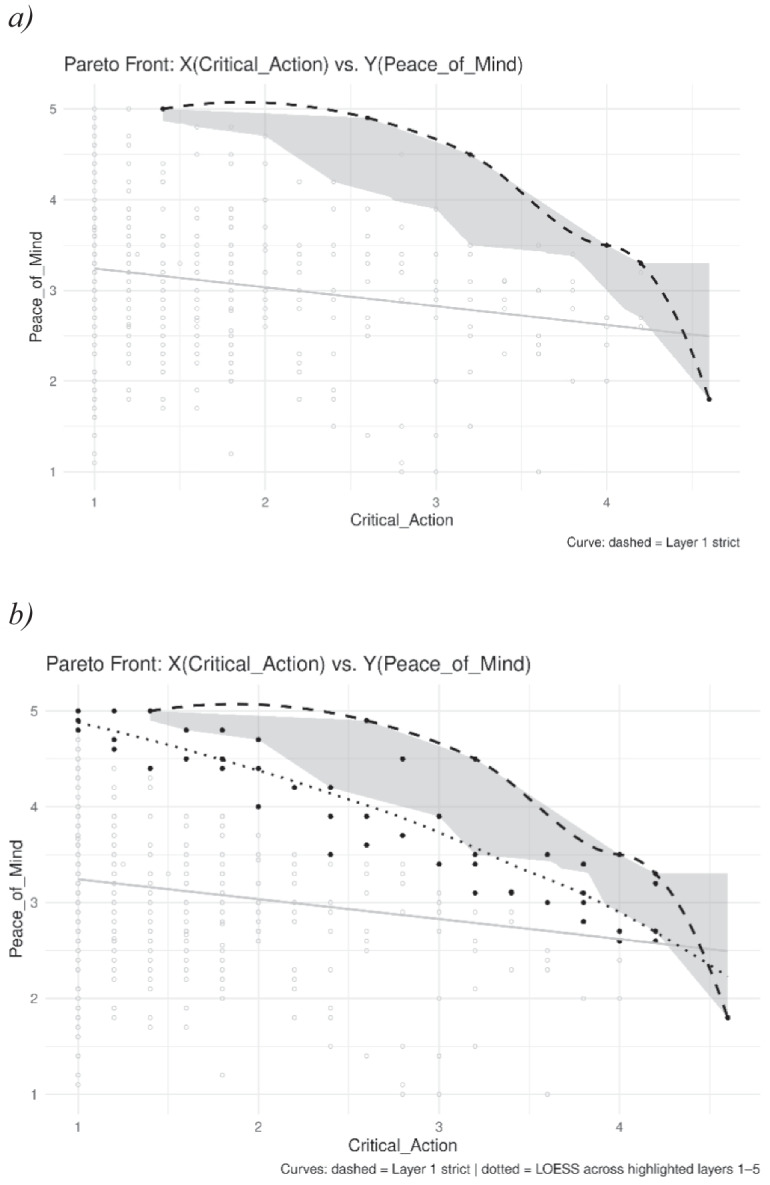

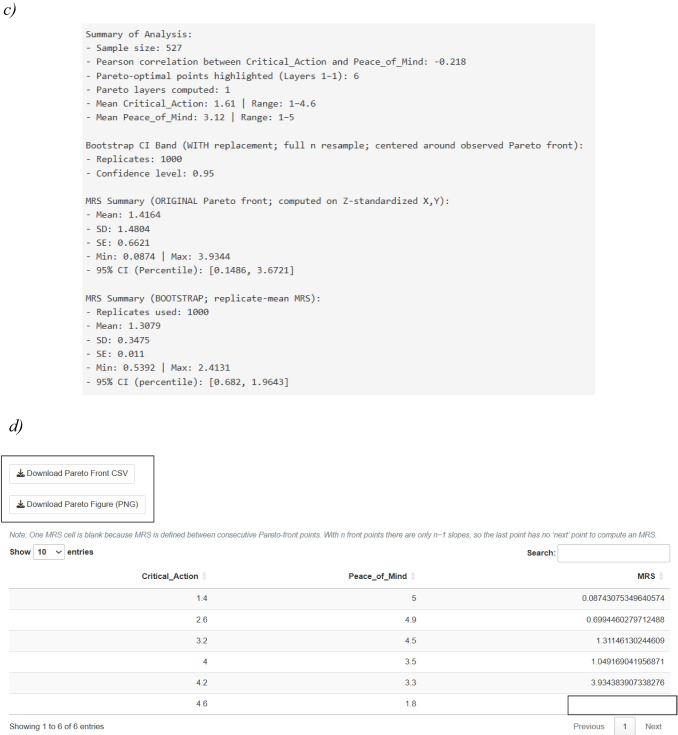
Optimization output from PO-Run. **a**) Dashed curve: Exact Pareto frontier (number of layers = 1). **b**) Dotted curve: Approximate frontier (number of layers = 5) **c**) Summary of PO analysis **d**) Summary of PO Analysis

We selected maximizing “critical action” and “peace of mind” as the two competing objectives for Pareto optimization in this illustration, set the number of Pareto layers as 1, and chose the default 95% CI and 1000 bootstrapping iterations. After running the analysis, Fig. 5a was first generated. This visualization contains four key elements: (1) all feasible solutions displayed as a scatterplot, (2) a regression line indicating the negative correlation between the two objectives, (3) the Pareto frontier represented by a dashed curve, and (4) a 95% confidence interval envelope surrounding the Pareto frontier.

To illustrate the function of configuring Pareto layers, Fig. 5b presents the same objectives but with five layers of solutions and shows a fitted trend that summarizes the broader tradeoff pattern. Notably, the layer configuration does not impact the MRS calculation or bootstrapping for the Pareto frontier, given that these results (shown in Fig. 5c) are based solely on the exact Pareto frontier (number of layers = 1). In practice, a quick way to distinguish between an exact and an approximate front is to check whether any solutions fall outside the frontier (i.e., in the impossible region). A Pareto frontier will have no black dots located in this region.

PO-Run also generates a panel of summary statistics (see Fig. 5c), including the valid sample size used in the analysis (e.g., *n* = 527), the correlation between the two objectives (e.g., *r* = –.218), the number of Pareto optimal solutions (e.g., *n* = 6), the means and ranges of the variables, and MRS statistics (both original and bootstrapped). All the Pareto optimal solutions are shown along with the output (see Fig. 5d), and can be downloaded as a CSV file, along with their IDs. This function is expected to be helpful for further analysis of only the Pareto-optimized data points, which may be substantively interesting. One MRS cell shows blank because MRS is defined between adjacent Pareto-front points. As aforementioned, with *k* Pareto optimal solutions, there are only *k*−1 discrete MRS_*i*_* or* segmental slopes (e.g., six Pareto optimal points yield five slopes in this example), so the last point has no next point with which to compute an MRS. Users can also download the generated visualization as a PNG file.

## Is there a plausible tradeoff between critical action and peace of mind?

It is important to note that PO-Run will always create a curve based on the available non-dominated solutions in the dataset. Thus, the mere presence of a curve in the output does not justify the existence of a tradeoff, and the plausibility of a tradeoff must be further evaluated by triangulating correlational evidence, theoretical basis, and other empirical evidence (e.g., visualization and MRS index) from the PO-Run.

In this example, there is a clear negative correlation between critical action and peace of mind, and furthermore, their tradeoff mechanism can also be theoretically justified. Specifically, research has shown that, although critical action is considered an ethically desirable attribute, it is nonetheless associated with poorer mental health outcomes (e.g., Maker Castro et al., 2022). Individuals with higher levels of critical consciousness are more likely to recognize various forms of social injustice (e.g., discrimination and microaggressions), and these experiences themselves can be significant sources of stress. Additionally, active engagement in sociopolitical movements for advancing social justice may further increase exposure to stressors. Desmarais and Christophe (2024) found that critical action exerted a stronger impact on adverse mental health outcomes (e.g., stress, anxiety, and depression) than other CC components (i.e., critical reflection and critical motivation). One possible explanation is that critical action does not guarantee desired outcomes (e.g., reducing social injustice), and the frustration of unmet goals can exacerbate stress.

Beyond correlational evidence and theoretical justification, we also evaluated the tradeoff through visualization and MRS results. As shown in Fig. 5a, a substantial impossible region appears in the upper-right corner of the plot. For example, no participants from the current sample simultaneously reported high levels of critical action (> 4.0 on the *X*-axis) and high levels of peace of mind (> 4.0 on the *Y*-axis). The bootstrapped confidence interval envelope (i.e., the gray band) for the Pareto frontier mostly falls under the curve, and only a small portion of the impossible region near the end of the *X*-axis could slightly shrink. This further confirms the substantialness of the impossible region. However, the frontier does not appear particularly sharp, as relatively few solutions lie near it, and most gray points cluster at a distance from the curve (i.e., low Pareto optimal solution tightness around the frontier).

We further examined the MRS to understand the rate and pattern of the tradeoff relations between the two optimization objectives. As aforementioned, the MRS represents how many SDs of the *Y*-axis objective must be sacrificed to gain one SD of the *X*-axis objective. The six non-dominated (Pareto optimal) solutions yielded five discrete MRS*ᵢ* values, ranging from.09 to 3.93, and the mean MRS_*frontier*_ was 1.42 (SE =.66, 95% CI = [0.15, 3.67]). It indicates that on average, each SD increase in critical action was associated with approximately a 1.42 SD decrease in peace of mind. Moreover, the tradeoff rate varied substantially across the frontier. There was almost no cost to peace of mind (MRS_1_ =.09; near a null tradeoff) when increasing critical action in the lower range of critical action. In contrast, the tradeoff rate was much higher (MRS_5_ = 3.93) near the higher levels of critical action, which means pursuing very high levels of critical action may require substantial sacrifice in mental health. This also echoes the very steep segmental slope near the right end of the Pareto frontier (see Fig. 5a). The bootstrapped MRS results provided supplemental information about stability of the results. Like the bootstrapped confidence interval envelope for the frontier, the bootstrapped MRS results did not alter our findings from the original sample.

Collectively, both the visualization and MRS index indicate a plausible tradeoff between the two objectives. The tradeoff rate varied across different levels of the two optimization objectives, as reflected by the range of MRS values observed within the current sample.

## Discussion

### Sample size and generalizability considerations for PO

As with most statistical analyses, results and conclusions from PO-Run are sample- and population-dependent. First, sample size has a direct impact on the stability of PO results. Inherently, the bi-objective PO analysis for tradeoffs extends on bivariate correlation, and the Pareto frontier is conceptually a representation of the boundary of observed scattered data points for two objectives in the optimization. Thus, the required sample size for achieving statistical stability in PO-Run should meet the sample guidelines for bivariate correlation. A widely recognized recommendation from Schönbrodt and Perugini (2013) suggests that a sample size of 250 is typically sufficient for stable correlational results, though alternative sample size decisions can be justified on a case-by-case basis with appropriate rationale. When the sample is insufficient, only a limited number of points would appear on the PO-Run plot, rendering the visualization less informative and potentially misleading (e.g., the appearance of a large impossible region attributable to sparse data rather than a genuine tradeoff relationship). The current illustrative example (*n* = 527), therefore, adequately satisfies the general sample size requirement for PO analysis. However, the stability of the MRS_frontier_ value might be questionable in the illustrative example, as this aggregation-based metric was calculated using only six optimal points (1.1% of the total sample). The bootstrapped statistics then provide supplemental information regarding the uncertainty of the MRS. Notably, the number of Pareto optimal points is not solely determined by overall sample size, and potential factors (e.g., measurement properties and experimental instructions) that can jointly determine the number of optimal points warrant a systematic investigation in the future. We elaborate on this nuanced issue further in the future direction section.

Moreover, the generalizability of findings to the broader population depends on the representativeness of the sample, and tradeoff relations may vary across populations. For instance, the present illustrative example is based on a sample of Asian and African Americans, and conclusions are therefore restricted to this particular population. In a different population, the tradeoff might look different. For example, clinical psychologists often receive extensive training in coping skills and stress management (Hannigan et al., 2004), which may buffer stress and alter the observed tradeoff. This highlights that tradeoffs can be conditional on the tested sample and population. Accordingly, we recommend that researchers explicitly define the population to which observed tradeoffs are inferred.

## Uses of PO-Run results

The available literature on psychological tradeoffs is relatively limited, so the question of how best to use PO results remains open. Here, we discuss several pathways (rather than prescriptive rules) for researchers to use PO results in both psychological research and applied settings, but we encourage researchers to adapt them to the specific context and demands of their own work.

### Using PO results in psychological research

The Pareto optimization analysis automatically yields a dichotomous variable indicating Pareto-optimal membership, that is, whether or not a given case falls on the Pareto frontier. Researchers can treat this variable as a dependent variable to investigate whether any factors or dimensions not originally included in the optimization nevertheless distinguish optimal from non-optimal cases. For example, is there a specific intervention in a psychological experiment that effectively motivates participants to approach Pareto-optimal performance (e.g., making decisions that are both rapid and highly accurate, Heitz, 2014)? Are participants with particular personality traits more likely to produce Pareto-optimal responses (e.g., impulsivity; Dickman & Meyer, 1988)? Further analyses (e.g., binary logistic regression) on Pareto optimization results can provide meaningful evidence for psychologists to address such questions and enrich our understanding of this psychological phenomenon.

When shifting focus from individual Pareto-optimal points to the overarching pattern of the Pareto frontier, researchers may also examine whether the frontier curve appears similar across various subpopulations. This effort can reveal a more precise picture of the tradeoff relationship between the constructs under study, which can support researchers in identifying subgroup differences and can also inform subsequent modeling decisions (e.g., whether multi-group modeling is warranted). Moreover, the PO method and results can complement other context-specific model-based approaches (e.g., evidence accumulation models [EAMs] in cognitive science; Boag et al., 2023; Ratcliff & McKoon, 2008) or intra-individual analysis to seek mechanistic explanations. Pareto analysis identifies where on the Pareto frontier a participant or condition falls, while EAMs further explain why and reveal potential intra-individual variation. Notably, these approaches are complementary in that they illuminate different aspects of the same phenomenon, though not necessarily convergent in findings, given that they (e.g., inter- vs. intra-individual analyses) may follow different methodological assumptions (Molenaar, 2004).

### Using PO results in applied psychological settings

In applied settings, PO results can inform the identification and selection of individuals for various purposes, which can be further refined to reflect the specific nature of psychological tradeoffs under study. Using the illustrative example of vicarious exposure to racism among racial minority communities, individuals who score high on both optimization objectives are those who actively engage in sociopolitical responses to social injustice without severe mental health costs. These are critical characteristics for certain roles, such as school counselors in underserved communities and community leaders advocating for social justice. Given the inherent tension between these two objectives, Pareto-optimal cases in such a context are comparatively rare (e.g., six out of 527 participants, about 1.1% in the example analysis in the current paper), which demonstrates the potential of PO as a selection tool, given that conventional screening methods would be unlikely to identify this profile efficiently.

As another example, PO results can also help select children for gifted programs. In Dumas et al. (2025), Pareto optimization analysis was used with elementary students to find those whose ideas best balanced originality and task appropriateness, the two core objectives of creativity that typically involve a tradeoff. Notably, the study showed that teacher ratings of student creativity only predicted optimized responses for students already identified for gifted or talented programs, indicating a possible halo effect that could disadvantage equally creative students who have not been identified. In contrast, the PO method identified the most creative responses more objectively, which indicates that PO could be a more useful tool for discovering highly creative students who might be missed by traditional gifted nomination methods.

## Future directions for methodological development

### A cross-validation framework for PO results

The current study has formulated both the conceptualization and the methodology for studying psychological tradeoffs. We have also developed and demonstrated the R Shiny application (PO-Run) for exploring this psychological phenomenon through Pareto optimization analyses, along with an array of empirical evidence (visualization, descriptive, and inferential statistics). Among these methodological developments, the bootstrap CIs for the Pareto frontier and MRS were designed to assess uncertainty within a single sample (i.e., internal precision), but they do not protect against overfitting the identified tradeoff to the current data. Cross-validating the tradeoff among psychological variables with external data or alternative research settings would be an important step for confirming the psychological phenomenon and enhancing its reproducibility (Yarkoni & Westfall, 2017), and this is particularly important for algorithm-driven optimization methods that usually pose a risk of overfitting (Dong & Dumas, 2025). As a future direction for advancing this method, it would be beneficial to establish a cross-validation framework that incorporates a collection of approaches (e.g., data partition and analysis techniques, machine-learning-based computational approaches, and sensitivity analyses) to establish the replicability of both the Pareto frontier and tradeoff pattern. Future integrations of these validation approaches into PO-Run would advance it from an exploratory tool to a comprehensive validation platform, which will substantially strengthen the evidential standards for psychological tradeoff research.

### Factors contributing to the number of optimal points

Another direction for PO research is to systematically investigate the factors that influence the number of optimal points observed. In the current illustrative example, despite a robust sample size (more than 500 valid cases), only a very small proportion of points (*n* = 6; 1.1% of the total sample) were identified as optimal, suggesting that the number is not solely determined by total sample size.

Other potential factors include measurement properties related to the observed variance of the objectives. Higher variance in optimization objectives allows data points to disperse more widely, which would theoretically create more opportunities for solutions to occupy distinct positions on the frontier without dominating each other (i.e., appearance of more optimal solutions). Generally, more response categories or scale options tend to yield greater variability in the objectives (e.g., Lozano et al., 2008). In the illustrative example, the *peace of mind* and *critical action* objectives were mean scores derived from five-point Likert scale items, which constitutes a relatively restrictive range compared to objectives with natural continuous metrics (e.g., response speed measured by task completion time), and the floor effect in the critical action variable (see Fig. 5a) may have exacerbated this limitation, ultimately resulting in only 1% of the sample being identified as optimal points. The restricted scale range may further compound this issue by increasing sensitivity to measurement error (Fife et al., 2012; Sackett & Yang, 2000), as scores compressed into a narrow band are more susceptible to random noise shifting solutions across dominance thresholds. This would potentially lower the reliability of classifying optimal and non-optimal points.

Beyond measurement properties, instructions (e.g., prompts) in psychological experiments have been shown to alter participants’ response patterns (e.g., task prompts for assessing creativity, Acar et al., 2020), potentially yielding varying numbers of optimal points. A prompt such as “try to be as accurate and as fast as possible in decision-making”, or “find ways to engage in critical action that also maintain your peace of mind” could encourage participants to pursue both objectives simultaneously, resulting in more points appearing on the Pareto frontier. Overall, considerable uncertainty remains regarding the emergence of optimal solutions, which warrants future research.

### High-dimensional PO for multivariate analyses

As noted above, the current study has explicitly defined psychological tradeoffs and introduced the Pareto optimization method for investigating this phenomenon. Accordingly, the current version of PO-Run is designed to be parsimonious in its application of bi-objective Pareto optimization. That said, Pareto optimization is fundamentally capable of accommodating multiple simultaneous objectives (Deb, 2011), and the approach can therefore be extended to richer multivariate research contexts in psychology (e.g., speed, accuracy, and cognitive load). Measurement research represents one particularly promising extension, as the psychometric properties of psychological scales (e.g., reliability, scale brevity, content coverage, and model fit) frequently exhibit tension with one another. Balancing these objectives can be challenging even for experienced psychometricians, so computational and algorithmic optimization methods for navigating these competing demands have seen growing adoption in this domain of psychological investigation (Dong & Dumas, 2025). Future methodological work may explore how Pareto optimization can be meaningfully integrated into measurement practices and other multivariate analytic frameworks.

## Conclusion

This study reviewed the conceptual and methodological foundations of Pareto optimization and developed PO-Run, an R Shiny application for exploring and analyzing plausible tradeoffs among psychological constructs. We also adapted the MRS index from econometrics to quantify psychological tradeoffs and built it into PO-Run. The application will enable users to conduct PO analyses and generate informative visualizations and MRS estimates for evaluating tradeoffs. To demonstrate the use of PO-Run, we provided an illustrative example focusing on a plausible tradeoff between critical action and peace of mind—two potentially conflicting psychological constructs. This application is expected to assist psychologists in moving toward an accessible and low-effort exploration of potential tradeoffs. These efforts may enrich psychological theory by offering alternative and novel perspectives for interpreting commonly observed negative relationships.

## Data Availability

The PO-Run app can be accessed via https://paretooptimization.shinyapps.io/Pareto/. The data for the worked example presented here are stored on OSF: https://osf.io/6fbhu/.

## Footnotes

1 Mathematically, the frontier's shape is determined by the variation of the trade-off rate across objective values, though it is likely uncommon in psychological contexts to encounter a perfectly constant trade-off rate (visualized as a straight line).

2 While reverse coding is a common practice in psychological research, re-labeling a reversed construct could pose the risk of distorting the meaning of the original measure if it is not handled carefully.

3 The speed–accuracy example embedded in the app is based on a simulated dataset. It is intended solely to illustrate the data requirements and an example trade-off pattern. It should not be interpreted as empirical evidence of the relation between the two constructs. Additional notes for this example (e.g., simulation parameters) can be found via the project’s OSF link.

## Code availability

The annotated R codes for building the application were also stored on Open Science Framework (OSF): https://osf.io/6fbhu/.

## Declarations

### Open practices statement

The present study was not preregistered. The PO-Run app can be accessed via https://paretooptimization.shinyapps.io/Pareto/. Both the annotated R codes for building the application and the data for the worked example presented here are stored on Open Science Framework (OSF): https://osf.io/6fbhu/.
